# Widespread deep-sea microorganisms in subseafloor geochemical cycling

**DOI:** 10.3389/fmicb.2026.1790163

**Published:** 2026-04-15

**Authors:** Renee L. Hageman, Sven Le Moine Bauer, Ingunn H. Thorseth, Jo Brendryen, Tor Einar Møller, Réka Böröcz, Hannah R. Babel, Bjarte Hannisdal, Steffen Leth Jørgensen

**Affiliations:** 1 Centre for Deep Sea Research, Department of Earth Science, University of Bergen, Bergen, Norway; 2 Bjerknes Centre for Climate Research, Department of Earth Science, University of Bergen, Bergen, Norway

**Keywords:** environmental change, widespread microorganisms, deep-sea sediments, environmental monitoring, environmental perturbations, microbial community, redox gradient

## Abstract

The subseafloor biosphere is one of the largest ecosystems on Earth, hosting dense populations of microbial cells. Microbial activity is critical to the Earth's geochemical cycles of major elements such as carbon and nitrogen. Despite a growing knowledge of subseafloor microbial diversity, the function and environmental significance of the many uncharacterized lineages remain elusive, along with their importance to the Earth's geochemical cycles. Identification of key microorganisms involved in the cycling of major elements is needed for advancing our understanding of how deep-sea sedimentary microbes influence global climate. Here we use the machine learning technique decision tree to identify which microbial families in deep-sea sediments correlate with geochemical variability in oxygen, nitrate, ammonium, and divalent manganese. We analyzed 1,114 deep-sea sediment samples from 60 sites at water depths ranging from 1,050–10,902 m located along the Arctic Mid-Ocean Ridge (20), the Western North Atlantic Gyre (3), the mid-Atlantic ridge (2), the North-West Pacific Ocean (14), the South China Sea (10), and the South Pacific Ocean (11). Decision tree rule mining revealed deep-sea sedimentary microbial families that likely contribute to subseafloor ecosystem functioning worldwide through four main metabolic pathways: aerobic nitrification, facultative anaerobic heterotrophy, anaerobic ammonium oxidation, and anaerobic heterotrophy. These families may thus be relevant targets for cultivation experiments, representation of functional groups in Earth system models, and monitoring of environmental change in deep-sea sediments caused by environmental perturbations (e.g., deep-sea mining and declining ocean oxygen concentrations).

## 1 Introduction

Global element cycles are profoundly affected by microorganisms (Nealson, 1997; Cavicchioli et al., 2019). Yet, the importance of deep-sea sedimentary microorganisms to global element cycles remains unknown due to knowledge gaps in their characterization and quantitative contribution to geochemical cycles (D'Hondt et al., 2019). Improving our understanding of deep-sea sedimentary microorganisms is essential for predicting how environmental changes will affect geochemical cycles using Earth system models. Currently, the biogeochemical pathways implemented in these models are oversimplified to assess the influence of microbial response and feedback mechanisms to changes in biogeochemical cycles (Cavicchioli et al., 2019; Zakem et al., 2020). Therefore, there is a need to identify which microorganisms are actively participating in global cycles, understand the specific processes they influence, and quantify their impact. As an initial step, we aim to identify the widespread sedimentary microorganisms that are presumably active and assess the major cycles they influence.

It has been documented that sedimentary community assemblages strongly correlate with chemistry over sediment-depth, where the availability of oxygen is an important driver for changes in subseafloor microbial community assembly (Hoshino et al., 2020; Zhao et al., 2020b; Møller et al., 2022). The phyla *Chloroflexi, Thaumarchaeota*, and *Proteobacteria* have been found to be the main indicators of change in oxygen, which lead to the suggestion that microbial community assemblages is shaped by the redox state (Hoshino et al., 2020). Also, in the deep-sea Arctic and hadal sediments, the redox state has been shown to be the main factor influencing microbial community structure (Jorgensen et al., 2012; Schauberger et al., 2021), suggesting that this might be a key factor in a larger area of the sedimentary subseafloor.

Finding correlations between chemistry and specific microorganisms within a sedimentary community is challenging. The cryptic cycling of chemical constituents and the presence of many uncharacterized lineages cause difficulty in determining microorganisms' contribution to the ecosystem (Zhang et al., 2021). Metagenomics accelerates the identification of genomic capabilities in these uncharacterized lineages; however, linking such genomic reconstructions to the functioning of microbial communities and chemical dynamics remains computationally challenging.

In this study, we used supervised learning to find patterns of associations between microbial community structure and chemistry data from deep-sea sediments to identify the microorganisms that potentially contribute to the cycling of oxygen, nitrate, ammonium and divalent manganese. Here, we explore the use of the decision tree (DT) that is an explanatory machine learning technique. The DT uses logic through rule-based explanations that we use to establish the associations between microbial community and chemical concentrations. The DT can be applied to both numerical and categorical data, and can model both linear and non-linear relationships, thus providing a more general approach than methods restricted to linear relationships (Kubinski et al., 2022). We assess the widespread microorganisms' relevance to chemical variations with ordination and multivariate analysis. The results show a strong relation between the widespread microorganisms and chemical variations and are consistent with literature. Many of these microorganisms are largely uncharacterized in function, and much less is known about their impact on geochemical cycles. We discuss the knowns and unknowns of their metabolic activity and its impact on geochemical cycles in deep-sea sediments.

## 2 Materials and methods

### 2.1 Data

**Figure 1:**
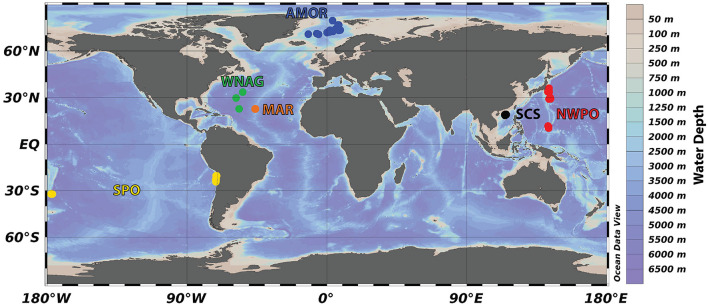
Locations selected in this study based on the availability of chemical and microbial data. The area acronyms and original studies are: Arctic Mid-Ocean Ridge (AMOR; this study; Zhao et al., 2020b; Møller et al., 2022), Western North Atlantic Gyre (WNAG; Vuillemin et al., 2020), Mid-Atlantic Ridge (MAR; Zhao et al., 2019), North-West Pacific Ocean (NWPO; Hiraoka et al., 2020), South Pacific Ocean (SPO; Schauberger et al., 2021), and South China Sea (SCS; Zhang et al., 2021). The map is generated with the Ocean Data View software (Schlitzer, 2022).

Geographical areas included in the analysis are the Arctic Mid-Ocean Ridge (AMOR; this study; Zhao et al., 2020b; Møller et al., 2022), Western North Atlantic Gyre (WNAG; Vuillemin et al., 2020), Mid-Atlantic Ridge (MAR; Zhao et al., 2019), North-West Pacific Ocean (NWPO; Hiraoka et al., 2020), South Pacific Ocean (SPO; Schauberger et al., 2021), and South China Sea (SCS; Zhang et al., 2021) (Figure 1). Of the 60 sites, 20 sites are located along the AMOR. The NWPO area includes 14 sites, one site was along the Japan Trench, 7 sites along the Izu-Ogasawara Trench, and 6 sites along the Mariana Trench. The SPO area has 8 sites at the Atacama Trench and 3 sites at the Kermadec Trench. The study at WNAG includes 3 sites, MAR 2 sites, and SCS 10 sites. The number of sites and samples, retrieval depth, and the sequencing data repositories are summarized in Table 1. These specific locations were chosen based on the availability of 16S rRNA gene data together with oxygen (O_2_), nitrate (${\text{NO}}_{3}^{-}$), ammonium (${\text{NH}}_{4}^{+}$), and manganese (Mn^2+^) pore fluid data.

**Table 1:** 16S rRNA data overview with accession numbers.

Location	Retrieval (meters below sea surface)	Number of sites/samples	NCBI accession number	Publication
Arctic Mid-Ocean Ridge (AMOR)	2,000 to 3,622	5/250	PRJNA784957 PRJEB102573	This study
Arctic Mid-Ocean Ridge (AMOR)	1,050 to 3,493	16/417	PRJNA529480 PRJNA789780	(Zhao et al. 2020b); (Møller et al. 2022)
Western North Atlantic Gyre (WNAG)	5,515 to 5,600	3/95	PRJNA590088 PRJNA473406	(Vuillemin et al. 2020)
Mid-Atlantic Ridge (MAR)	4,425 and 4,475	2/43	PRJNA489438	(Zhao et al. 2019)
North-West Pacific Ocean (NWPO)	4,700 to 10,902	14/91	PRJDB8106	(Hiraoka et al. 2020)
South Pacific Ocean (SPO)	4,050 to 10,010	11/84	PRJEB33873	(Schauberger et al. 2021)
South China Sea (SCS)	1,250 to 3,530	10/134	PRJNA606622	(Zhang et al. 2021)
Total		60/1114		

The data collected in this study covers 250 sediment samples from AMOR retrieved from the seabed, where water depths ranged from 2,000–3,622 m. These sediment samples came from 5 cores, GS19GC05 (74°14.186'N, 05°29.653'E), GS19GC10 (73°37.676'N 07°49.398'E), GS21GC07 (72°45.363'N, 03°49.634'E), GS21GC08 (72°45.324'N, 03°49.979'E), and KH22GC02 (79°36.700'N, 03°39.236'E). Immediately after retrieval, the cores were sectioned into working and archive halves and oxygen was measured with a micro-fiber optic oxygen transmitter (Microx TX3, PreSense). Simultaneously, the cores were sub-sampled on board for both the geochemistry and microbiology from the working half. The pore fluid was extracted with Rhizon sampler (Rhizosphere research products, Denmark) using sterile 20-mL syringes, whereas the sediment was carefully collected using either a cut-off top 10-mL sterile syringe with a diameter of 1 cm or sterile spatula and stored at –80 °C.

### 2.2 Pore fluid analysis

The pore fluid samples were aliquoted for later analyses of cations and nutrients. Samples for cation analysis were filled in acid-cleaned HDPE bottles, acidified with ultrapure HNO_3_ to a final concentration of 3% v/v, and stored at 4°C until analysis by a Thermo Scientific iCAP 7600 inductively coupled plasma optical emission spectrometer (ICP-OES). Samples for nutrient analyses (i.e., ammonium and nitrate) were stored at –20°C until analyzed by photospectrometry using a Seal Analytical Quaatro continuous flow analyzer.

### 2.3 ^14^C analysis and age model generation

The GS19GC10 core was dated using six samples of the planktonic foraminifera *Neogloboquadrina pachyderma* extracted at 15, 28, 38 78, 115, and 165 cm, and analyzed at the Radiocarbon Dating Laboratory (Lunds Universitet, Sweden) using accelerated mass spectroscopy. The ^14^C dates and stratigraphic information were subsequently combined into a Bayesian age model consisting of 4224 independent realizations using OxCal (v.4.4, Ramsey, 2008, 2009, Figure 4B). The ensemble is convergent, meaning that adding another realization will not significantly alter the probability distribution of the existing set. Each realization is a vector of certain, non-decreasing elements resulting from a Poisson process along increasing sediment depth constrained using a naive Bayesian classifier with the P_Sequence and variable k options (Ramsey, 2008). Because our framework requires strictly increasing elements in the age model (i.e., a sample deeper than another one has to be older), infinitesimals that would not impact downstream analysis were added wherever necessary to remove effects of machine rounding and non-increasing element sequences.

### 2.4 DNA extraction and library preparation

In this study we extracted DNA and performed 16S rRNA gene amplification of the V4 region from the cores GS19GC05, GS19GC10, GS21GC07, GS21GC08, GS21GC09, and KH22GC02. The DNeasy PowerLyzer PowerSoil Kit (Qiagen) was used for DNA extraction following the manufacturer's instructions with modifications in step 4, where we chose to use the MP fastprep 24 for 45 second at 5 m s^−1^, and step 10, where we transferred 650 μL instead of 750 μL. Additionally, extraction blanks were included with each batch and processed through sequencing with samples. To cover the *Bacteria* and *Archaea* domains, universal prokaryotic primers were used: 519F (5'-CAGCMGCCGCGGTAA-3') and 805R (5'-GACTACHVGGGTATCTAATCC-3') (biomer.net, Germany). Resulting amplicons were sequenced on an IonTorrent Personal Genome Machine in the Laboratory of Biodiversity, University of Bergen, Norway.

### 2.5 Sequence processing

The sequences were processed through an adapted VSEARCH pipeline (Rognes et al., 2016; Le Moine Bauer et al., 2023) and taxonomically classified using the CREST algorithm (Lanzén et al., 2012) applying the Lowest Common Ancestor algorithm with SILVA (version 138pr2; Yilmaz et al., 2014) as the reference database. Each study was run separately through the pipeline and taxonomic classification. The operational taxonomic unit (OTU) tables were combined using phyloseq (version 1.38.0; McMurdie and Holmes, 2013) package in *R* (version 4.1.2). To avoid agglomerating unrelated OTUs, we manually checked taxonomic assignments for abundant OTUs that were classified only at the superkingdom level. We identified 506 archaeal OTUs whose taxonomy was resolved no further than “Archaea superkingdom”, that includes one highly abundant OTU. We reclassified all 506 OTUs in the ARB-SILVA database (www.arb-silva.de/aligner) that resulted in the most abundant OTU being classified as *Lokiarchaeota* and several unclassified assignments for lower abundant OTUs. We then compared the dominant archaeal OTU (reclassified as *Lokiarchaeota*) with the 505 OTUs. OTUs were merged with *Lokiarchaeota* when they showed 100% sequence overlap and ≥97% identity to the dominant OTU.

The blanks were inspected to identify contamination. The blanks consist of cross contamination during DNA extraction and amplification, therefore, not every taxon can be removed. Taxa were removed when identified as contaminants without marine origin or as lab contaminants identified by (Eisenhofer et al. 2019).

### 2.6 Data analysis

The analysis requires pore fluid measurements for each microbial data point. Since measurements are not available for all depths at the microbial sampling intervals, we linearly interpolated pore fluid depth profiles for each core using the interpolate function from the SciPy package (version 1.10.0; Virtanen et al., 2020) in *Python* (version 3.10.12). Linear interpolation was chosen because it introduces the least assumptions compared to other interpolation methods. Additionally, we included the anoxic samples of SCS sites in the analysis by assigning 0 μM [O_2_] to samples with [NH_4_] ≥ 10 μM and the presence of manganese covering 96 samples (Table 2).

**Table 2:** Number of samples available for analysis per site and chemical constituent.

Site	O_2_	NO_3_	NH_4_	Mn
Arctic Mid-Ocean Ridge (AMOR)	628	629	629	644
Western North Atlantic Gyre (WNAG)	95	0	0	0
Mid-Atlantic Ridge (MAR)	41	43	0	0
North-West Pacific Ocean (NWPO)	67	91	91	0
South Pacific Ocean (SPO)	37	71	61	73
South China Sea (SCS)	96	134	134	134
Total	964	968	915	851

While using relative abundance 16S rRNA gene and chemical concentration data, we followed the principles of compositional data analysis. Both data types are compositional as they describe parts of a whole, where relative abundance is constrained to sum to one, and concentrations are constrained by liquid volume (Pawlowsky-Glahn et al., 2015). Therefore, we adhere to these principles and consider compositionality by using the Aitchinson distance, in which a center log ratio (CLR) transformation is applied to the compositional data (Aitchison, 1982; Pawlowsky-Glahn et al., 2015; Gloor et al., 2017). CLR transformation requires all values to be above zero, therefore, the value one was added to all OTU counts and 0.001 μM to the chemical concentrations. This zero-imputation method has been shown to be better than other methods for data with a high number of zeros (Lubbe et al., 2021).

The DT machine learning approach was used to predict chemical data (i.e., oxygen, nitrate, ammonium, and divalent manganese) from microbial community assembly. The table with microbial community assembly (OTU table) consisted of ~144,000 OTUs and the number of samples ranged from 851–964 depending on the predicted chemical variable (Table 2). To make the method applicable to our dataset, the OTU table data requires a reduction in the number of dimensions, that are the number of OTUs, since there are more decisions to be made than observations to be learned from (Liu et al., 2005). The first dimensionality reduction was done by binning the OTUs on family level using the phyloseq package in *R*, resulting in 1342 families. The number of families was still higher than the number of samples causing that the number of samples remained insufficient to converge the model in selecting important rules (Liu et al., 2005). To further reduce dimensions, we selected taxa that showed significantly different relative abundance between absence and presence of at least one chemical constituent (i.e., oxygen, nitrate, ammonium, and manganese). To test the significance, we calculated p-values that were corrected to diminish the false discovery rate using Storey's q-value method (Storey and Tibshirani, 2003). The application of Storey's method resulted in 209, 120, 199, and 125 significant taxa for oxygen, nitrate, ammonium, and manganese, totalling 425 unique taxa.

**Figure 2:**
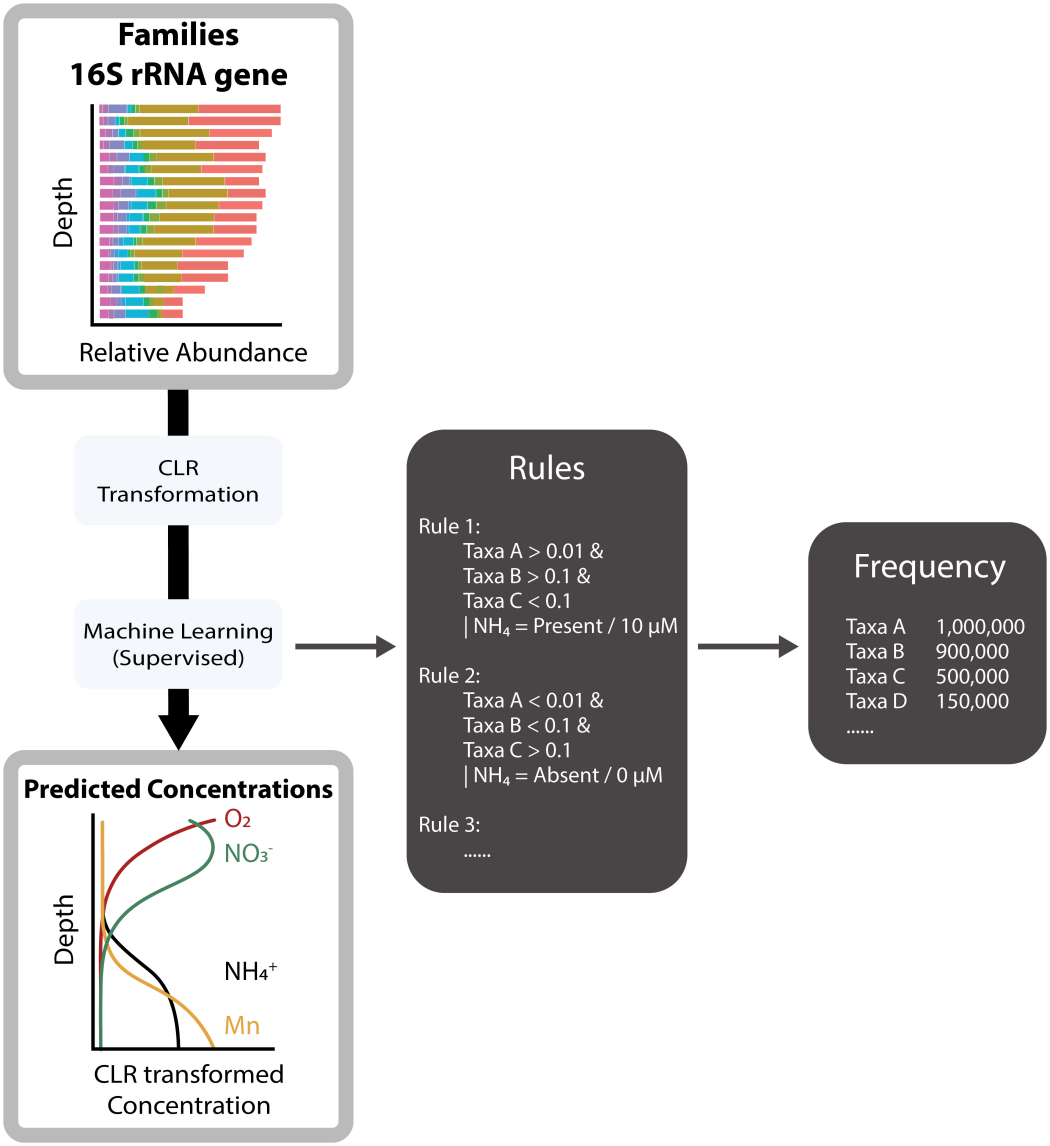
Method overview for retrieving the most important taxa. The input variable is the clr-transformed relative abundance data predicting the CLR-transformed concentrations of oxygen, nitrate, ammonium, and manganese.

The CLR transformed relative abundances of the 425 taxa were used to predict continuous CLR transformed oxygen, nitrate, ammonium, and manganese concentrations and presence and absence of chemical constituent using the supervised machine learning techniques DT regressor and classifier (Figure 2) from the scikit-learn package (version 1.2.1) in *Python*. DT regressor is used to predict continuous target variables using sum of squared deviation from the mean for data splitting and DT classifier predicts categorical target variables using gini impurity index (Loh, 2011). DT was chosen for its potential ability to explain predictions, unlike alternative machine or deep learning methods. Additionally, DT is a non-parametric method (Murthy, 1998) that can detect non-linear including non-monotonic relationships (Dhebar et al., 2024), which are required for finding relations in non-linear microbial data (Kubinski et al., 2022). The combination of CLR transformation and DT methods have shown to be effective and robust in handling complex data with non-linear features (Kubinski et al., 2022; Gao et al., 2025).

The explanations are expressed in rules that are formed by the DT algorithm (Figure 2), where the rules can be used to identify taxa explaining variations in the data (Chen et al., 2014; Cao et al., 2021; Rudar et al., 2023). The rules were being used to infer the importance of taxa. The importance was derived from the frequency of a taxon used for decisions that lead to predicting the concentrations. The higher a taxon is in the tree of decision making, the more important the taxon is. The most important taxa were determined over 1,000 independent iterations of data splitting and DT training. The DT was trained using Monte-Carlo cross-validation, where the dataset was split in a cross-validation (70%) and test (30%) set. The DT forms the rules on the cross-validation dataset and then uses these rules to predict the test dataset to obtain the accuracy of the model. We selected what we refer to as the “widespread taxa” based on two criteria (1) the frequency of a taxon used by the DT to make a prediction of chemical concentrations. The higher the frequency the higher its assumed importance for predicting the chemical concentration. (2) The taxon should be present across all geographical areas. The taxa with the highest frequency, which also were present across all geographical areas, we then selected as part of the 20 widespread taxa. The widespreadness is further discussed in Section 3.2.

We used compositional data principal component analysis (CoDa-PCA) to quantify how much of the dataset variation is explained by the widespread taxa (Aitchison, 1982; Pawlowsky-Glahn et al., 2015). Additionally, we used distance-based redundancy analysis (Legendre and Anderson, 1999, db-RDA) to assess how much of the geochemical variations explains the variation in widespread taxa (Dixon, 2003, rda function from VEGAN; version 2.6-4)

The code and data used to generate the figures in this manuscript are available on GitHub, NCBI, and PANGAEA. The references can be found in the data availability statement.

## 3 Results and discussion

In the Results and Discussion section, we will provide an overview of the model's performance in predicting chemical variations using the selected taxa, and how these taxa relate to chemical variations (Sections 3.1 and 3.2). Subsequently, we will examine whether these observations align with findings from literature and the knowledge gaps (Sections 3.3–3.6). Finally, we will discuss the implications of our results on the survival of strictly anaerobic microorganisms in deep-sea sediments (Section 3.7), the relevance of the metabolisms to global geochemical carbon cycling (Section 3.8), and consequences of environmental perturbations to the widespread community (Section 3.9).

### 3.1 Widespread taxa associated with geochemical variations

To identify the widespread taxa in the deep biosphere we applied the DT models that formulate rules to generalize patterns. We applied DT to explain the dependency of community assemblages to chemical variations in oxygen, nitrate, ammonium, and manganese. The training data consist of microbial community assemblages and chemical data from deep-sea sediments worldwide (Figure 1). The generalization over a worldwide dataset provides widespread taxa involved in changing concentration of oxygen, nitrate, ammonium, and manganese globally.

The DT found associations between microbial community assemblages and concentrations of oxygen, nitrate, ammonium, and manganese with a *p*-value < 0.05 as shown in Supplementary Figure S1. These results were consistent across methods, as both the regressor and classifier DT achieved similar accuracy (Supplementary Figure S2) and selected the same taxa. Model accuracy was influenced by sample size, where larger sample sizes yielded higher accuracy (Supplementary Figures S3, S4). While decision trees can express non-linear dependencies between taxa via explicit if–then rules, the learned rules may be overly complex. The rules cannot be directly interpreted biologically, similarly to the more familiar linear method, PCA, but the method can be used to identify the most important features or loadings explaining the structure of the data. Therefore, we are limited to identifying the most important taxa that explain the variations in redox sensitive constituents.

**Figure 3:**
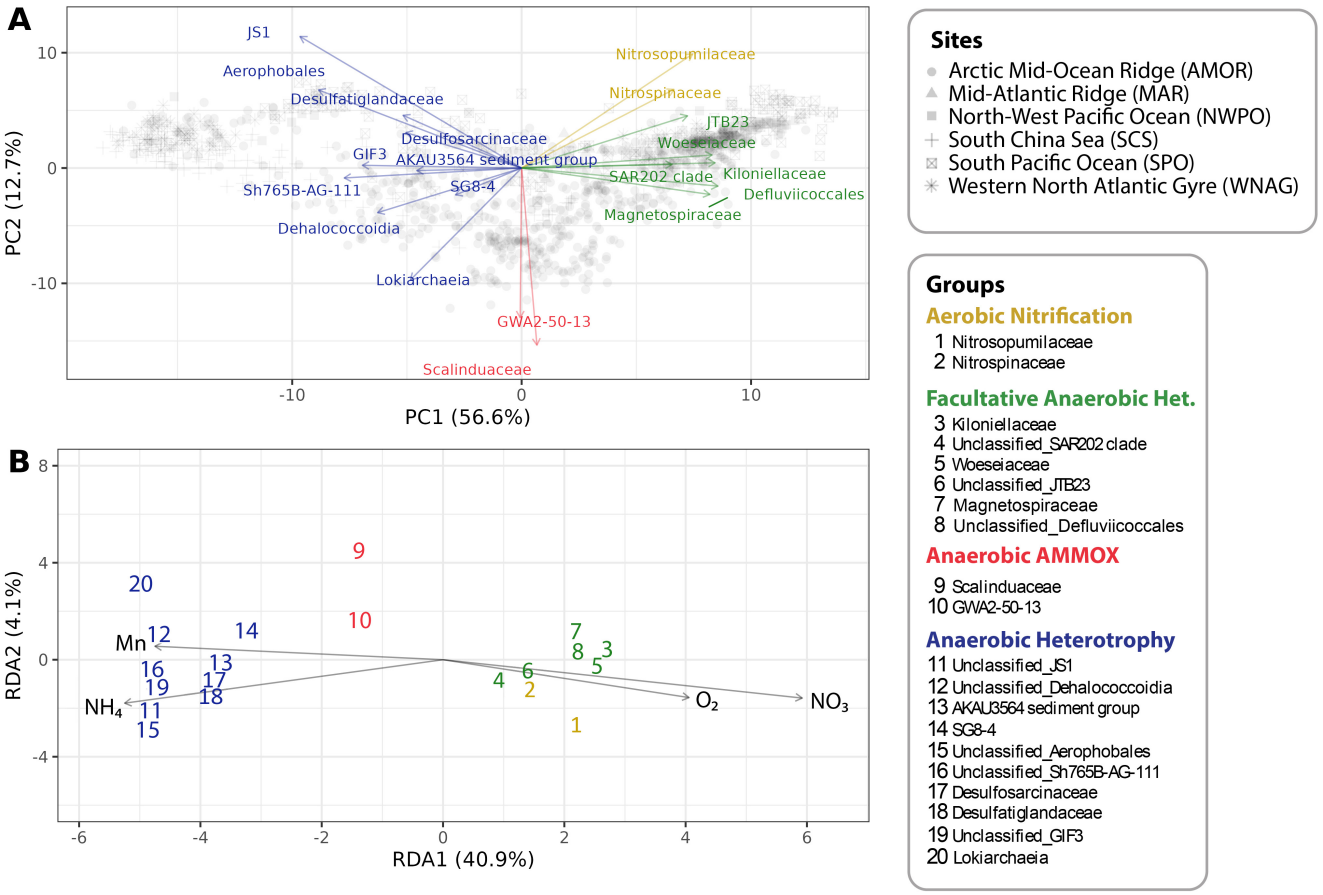
CoDa-PCA and db-RDA display the globally important taxa that are color coded based on inferred metabolic capabilities. **(A)** Two dimensional CoDa-PCA biplot explaining 69.3% of the data variance by the selected widespread taxa. **(B)** db-RDA plot of the the linear relation between the important taxa and the concentrations of oxygen, nitrate, ammonium, and manganese. Both plots show highest variance on the x-axis that is explained by the redox potential, where high redox potential is signified by high [O_2_] and low redox potential by high divalent [Mn] or more reduced compounds.

The selected 20 widespread taxa (loadings) formed relatively distinct clusters along PC1 and PC2 in the ordination space (Figure 3A). In addition to PCA, db-RDA analysis allowed us to establish the linear relation between the variance of chemical constituents and the widespread taxa. Overall, PC1 and PC2 explained 69.3% of the variation in the data and RDA1 and RDA2 explained 45.0% of the variance among the 20 widespread taxa.

Global quantitative geochemical and widespread taxa variability resulted in four clusters of taxa with similar metabolic capacities within each cluster (Figure 3). The clustering is in accordance with observational data and characterized taxa in literature (Sections 3.3–3.6). The yellow cluster represents aerobic nitrification, including *Nitrosopumilaceae* and *Nitrospinaceae*. The green cluster is represented by facultative anaerobic heterotrophy group containing *Kiloniellaceae, SAR202* (*Dehalococcoidia* class), *Woeseiaceae, JTB23* (*Gammaproteobacteria* class), *Magnetospiraceae*, and *Defluviicoccales*. The red cluster includes *Scalinduaceae* and *GWA2-50-13* (*Bathyanammoxibiaceae*; Section 3.5), known for anaerobic ammonium oxidation (anammox). Finally, the blue cluster consists of anaerobic heterotrophs, primarily the class *Dehalococcoidia* (orders *GIF3, Sh765B-AG-111*, and other unclassified *Dehalococcoidia*), along with sulfate-reducing families *Desulfatiglandaceae* and *Desulfosarcinaceae* belonging to the class *Desulfobacteria*. Additional members include *AKAU3564 sediment group* and *SG8-4* (phylum *Planctomycetota*), as well as the class *Lokiarchaeia, JS1* (*Atribacteriota* phylum), and the order *Aerophobales*. This clustering highlights key metabolisms contributing to global geochemical cycles.

**Figure 4:**
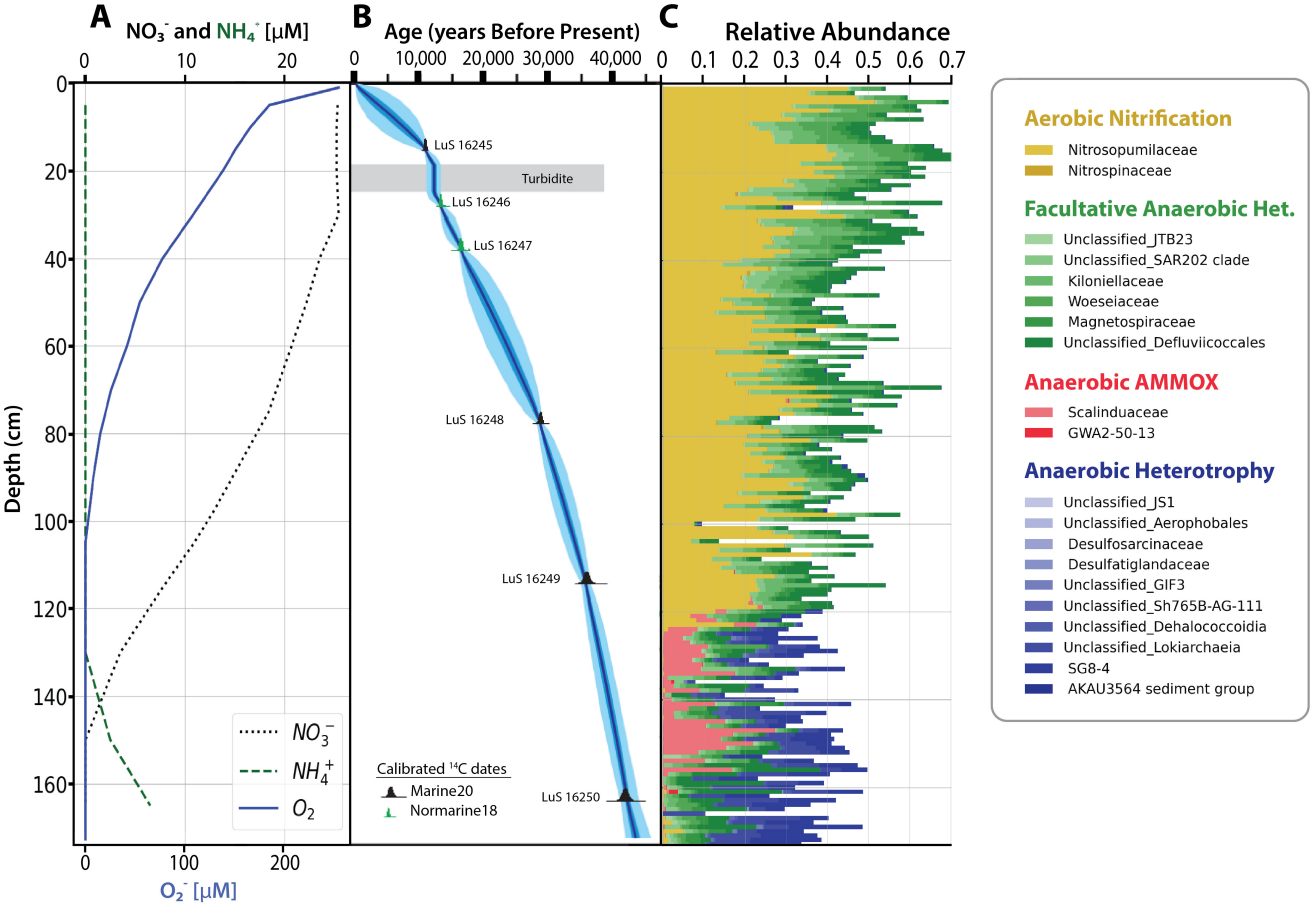
The GS19GC10 sediment depth profile of **(A)** oxygen, nitrate, and ammonium concentrations, **(B)** age model generated using OxCal., and **(C)** relative abundance of the four groups.

The difference between oxic and anoxic explains most of the variance of these taxa (RDA1 40.9%), where oxic is defined by high concentrations in oxygen and nitrate, and anoxic by absence of oxygen and presence of ammonium and often manganese. Because taxa separate along the RDA1 axis in the same way as along PC1, this suggests that both axes primarily explain variation driven by redox potential. In sediments, the redox potential is highest at high oxygen concentrations and declines progressively as electron acceptors are depleted, beginning with oxygen, followed by nitrate, and so forth. The aerobic nitrifiers and facultative anaerobic heterotrophs are present in oxic environments. Anammox and anaerobic heterotrophs are more abundant under slightly to very reduced anaerobic conditions, represented by hypoxic to anoxic conditions that are signified by increasing ammonium and manganese concentrations (Figure 3). In core GS19GC10, where centimeter resolution sampling was performed, a profound shift in microbial community structure occurs around 120 cm, where the anaerobes transition from rare to common as oxygen is depleted and ammonium appears (Figures 4A, C).

The DT analysis focuses on common taxa that significantly respond to changes in redox sensitive constituents, as these may be most relevant to environmental change. Presumably, other microorganisms also contribute to the cycling of these constituents, but are not considered in this study. Additionally, the analysis is limited by primer bias that potentially excludes certain groups which might contribute to changes in chemistry. Such bias can reduce model accuracy by missing essential microorganisms that significantly affect concentration profiles.

### 3.2 Global distribution

**Figure 5:**
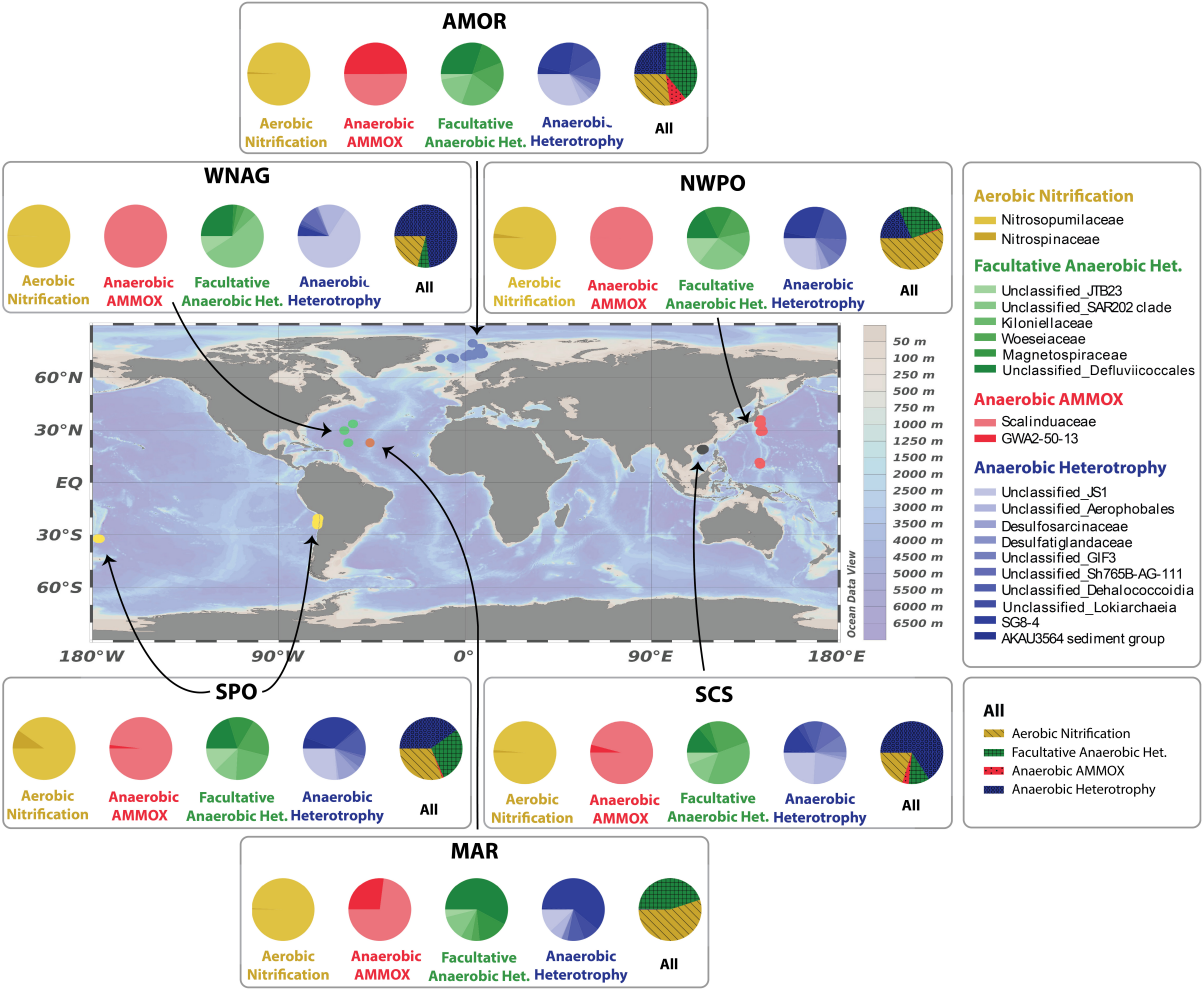
Distribution of widespread deep-sea microorganisms. The first four pie charts represent the sub-composition of the four metabolic groups. The last pie chart (All) displays the sub-composition of the metabolic groups with respect to each other.

The distribution of samples taken over depth changes for each location causing an unequal number of samples in the oxic, oxic-anoxic transition, and anoxic zone. These unequal sample numbers from oxic, oxic-anoxic transition, and anoxic zones are reflected in the relative abundance of each metabolic group per location (Figure 5 All) and influences the selection of taxa. The oxic zone is primarily shown by the aerobic autotrophs, the transition zone by anammox, and the anoxic zone by anaerobic heterotrophs. The oxic zone has numerous samples at every site, whereas the transition from oxic to anoxic is less represented at each site by spanning only a few centimeters over depth. The overall representation of transition zone samples can be seen by the anammox taxa that are primarily located near the transition zone at low oxygen concentrations (Figure 3) because of their metabolic need for chemical constituents primarily available under hypoxic conditions (i.e., nitrite and ammonium; Jetten et al., 1998). The transition zone is more thoroughly sampled in the AMOR site, resulting in higher relative abundance of anammox taxa. The anoxic site is more represented in WNAG (Figure 5 WNAG All, Supplementary Figure S6) and SCS (Figure 5 SCS All, Supplementary Figure S8) which can be observed by the higher relative abundance of anaerobic heterotrophs. MAR barely consists of anoxic samples and anaerobic heterotrophs (Figure 5 MAR All, Supplementary Figure S7) due to oxygen ventilation from the basin causing a C-shaped oxygen concentration profile over depth (Orcutt et al., 2013; Edwards et al., 2014). This leads to biases in the selection procedure and can cause overlooked taxa that are of importance to the cycling of oxygen, nitrate, ammonium, and manganese.

The distribution of taxa shows a difference between the number of selected autotrophs and heterotrophs. The autotrophs are primarily dominated by one family over all sites, where the aerobic autotrophs are dominated by *Nitrosopumiales* and the anaerobic autotrophs are dominated by *Scalinduaceae*. *GWA2-50-13* (*Bathyanammoxibiaceae*; Section 3.5) is more abundant in AMOR and MAR than the other sites. For the heterotrophic groups there is no clear dominating family, however, more families are selected than for the autotrophic groups.

### 3.3 Aerobic nitrification

Nitrification is a dual reaction involving ammonium oxidation and nitrite (${\text{NO}}_{2}^{-}$) oxidation that are catalyzed by ammonium-oxidizing bacteria (AOB) or archaea (AOA) and nitrite-oxidizing bacteria (NOB). The two-step nitrification reaction can lead to a cross-feeding mechanism of AOB/AOA and NOB (Könneke et al., 2005). Our findings show close relation between relative abundance distributions of *Nitrosopumilaceae* (AOA) and *Nitrospinaceae* (NOB) (Figure 5, Supplementary Figures S5–S10).

*Nitrosopumilaceae* is an archaeon that oxidizes ammonium to nitrite (Martens-Habbena et al., 2009). We find this archaeon dominantly present at all sites in the oxic zone (Supplementary Figures S5–S10) and under anoxic conditions with the availability of nitrate/nitrite and ammonium. Under anoxic conditions *Nitrosopumilus maritimus* has been suggested to perform nitric oxide (NO) dismutation that produces nitrogen gas and oxygen to continue the oxidation of ammonium (Kraft et al., 2022), which could explain the occasional increased abundances observed when oxygen is depleted (Supplementary Figures S5, S9). The *Nitrosopumilaceae* family has been reported in many deep-sea sediment studies as highly abundant (Zhao et al., 2020a; Sun et al., 2022; Zhang et al., 2023; Sun et al., 2023) and has been observed as more abundant in deeper waters (Zhao et al., 2020a; Shi et al., 2023) and even found in deep-sea sponges (Garritano et al., 2023).

The NOB *Nitrospinaceae* is involved in nitrite oxidation (Watson and Waterbury, 1971) and our results reveal higher abundance under lower oxygen concentrations (10–140 μM; Supplementary Figures S5–S10). *Nitrospinaceae* have been abundantly observed mainly in marine sediments and oxygen minimum zones (Ngugi et al., 2016). Through metagenomic information, several *Nitrospinia* have shown the potential to perform nitrite oxidation and have been suggested to prefer lower oxygen environments (Fortin et al., 2024). The prevalence under lower oxygen concentrations might be related to the increase of nitrite during oxygen depletion, promoting nitrite oxidation.

### 3.4 Facultative anaerobic heterotrophy

The facultative anaerobic heterotrophs group consists of taxa that are active in respiration using organic carbon as electron donor. Some of these taxa have shown to be able to switch between aerobic and anaerobic metabolism. Therefore, this group is called facultative anaerobic heterotrophs. The widespread group consists of the phyla: *Alphaproteobacteria* (*Kiloniellaceae, Magnetospiraceae*, and an unclassified *Defluviicoccales*), *Gammaproteobacteria* (*Woeseiaceae* and *JTB23*), and *Chloroflexota* (*SAR202*). The members of this group were found in oxic and hypoxic environments (Figure 3B, Supplementary Figures S5–S10).

*Kiloniellaceae* is a facultatively anaerobic chemoheterotroph with nitrate as electron acceptor (Wiese et al., 2009; Wang et al., 2015). We found its prevalence in oxic environments (Figure 3B, Supplementary Figures S5–S10). In the case of *K. laminariae*, nitrate is reduced to nitrous oxide (N_2_O) (Imhoff and Wiese, 2014) and is unable to oxidize ammonia (Wiese et al., 2020). Additionally, the strains *K. laminariae* and *K. antartica* have been shown to perform complete denitrification pathway from nitrate reduction to the production of dinitrogen gas (Si et al., 2017).

The selected unclassified *Defluviicoccales* family is, aside from their heterotrophy, not well described (Maszenan et al., 2022; Wang et al., 2014). Metagenomic and enrichment batch reactor studies showed that *Defluviicoccus* might be capable of nitrate reduction to nitrite, where dissimilatory denitrification was not encoded (Onetto et al., 2019; Wang et al., 2008, 2014). In addition, *Defluviicoccus* has been suggested to perform nitrite reduction to nitrous oxide (Wang et al., 2020). However, more research is needed to reveal the detailed denitrifying capacity of *Defluviicoccales*.

One strain from the *Magnetospiraceae* family (*Rhodospirillales*) has been cultured, namely *Magnetospira thiophila*. This strain is known to be capable of chemoorganoheterotrophic and chemolithoautotrophic growth that thrive in the oxic-anoxic transition zone (Williams et al., 2012). The strain was able to use oxygen as electron acceptor under heterotrophic conditions. During autotrophy, the strain uses thiosulfate as electron donor and nitrous oxide acts as electron acceptor (Williams et al., 2012). This strain has polar magnetotaxis (Williams et al., 2012) that uses the magnetic field as direction for migration (Frankel et al., 1997). More distantly related species to the family *Magnetospiraceae* (Williams et al., 2012), *Magnetospirillum gryphiswaldense*, performs dissimilatory nitrate reduction to ammonium and denitrification (Li et al., 2012; Hu et al., 2022). In addition, both pathways contribute to the formation of magnetite to establish magnetotaxis (Bazylinski et al., 1988; Schüler and Frankel, 1999; Li et al., 2012). *Magnetospiraceae* has been reported on polymetallic nodules and Indian hydrothermal systems (Adam-Beyer et al., 2023) and associated with potential metal cycling capacities (Shulga et al., 2022).

The gammaproteobacterial *Woeseiaceae* is a heterogeneous family that covers facultative sulfur- and hydrogen-based chemolithoautotrophy metabolism up to obligate chemoorganoheterotrophy (Mußmann et al., 2017). The family *Woeseiaceae* is ubiquitous in deep-sea sediments (Hinger et al., 2019; Hoffmann et al., 2020) with high relative abundance in oxygen and nitrate-rich sediments, as shown in our findings in Supplementary Figures S5–S10. The taxa previals in tidal, estuary, and deep-sea sediment systems (Mußmann et al., 2017). The only cultured strain *Woeseia oceani* dominates deep-sea sediments (Hoffmann et al., 2020), and has shown to be an oxygen respirating obligate chemoorganoheterotroph with genetic markers encoding nitrite and nitric oxide reduction to nitrous oxide production (NirS, NorB; Mußmann et al., 2017). There is evidence that *W. oceani* produces nitrous oxide in culture (Hinger et al., 2019). Therefore, *Woeseiaceae* might play an important role in the nitrogen cycle.

*JTB23* is a *Gammaproteobacteria* that is observed in surface sediments (Rubin-Blum et al., 2022); however, its metabolism remains unknown. Figure 3B indicates the dependence on oxidized environment with most similar trends to *SAR202* clade and *Woeseiaceae*, suggesting chemoorganoheterotrophy using oxygen and/or nitrate as electron acceptor.

The heterotrophic *SAR202* clade is known as a facultative anaerobe consisting of versatile metabolic capabilities (sulfite, nitrate, and nitrite reduction; Mehrshad et al., 2018; Wei et al., 2022; Lim et al., 2023). All metabolic capacities were shown active through transcriptomics, depending on which sub-cluster of *SAR202* clade (Wei et al., 2022). There is no conclusion on what type of metabolism *SAR202* clade has in deep-sea sediments. Yet, our result show that *SAR202* clade has a similar distribution as *JTB23* (Supplementary Figures S5–S10), which might suggest a similar electron acceptor.

Taxa within the facultative anaerobic heterotrophic group are likely important contributors to geochemical cycling. These taxa are of interest for further investigation to elucidate their biogeochemical pathways, through cultivation or isotopic labeling. The functions of the *SAR202* clade and the orders *Defluviicoccales* and *JTB23* are not well established in literature and requires further investigation to clarify their roles in geochemical cycles. While the facultative anaerobic heterotroph families *Magnetospiraceae* and *Woeseiaceae* are more investigated, their specific function in deep-sea sediments are still not well constrained, making it difficult to verify their contributions to specific geochemical processes.

### 3.5 Anaerobic ammonium oxidation

Anammox bacteria are obligate anaerobes coupling the oxidation of ammonium to the reduction of nitrite producing dinitrogen gas (Jetten et al., 1998).

*Scalinduaceae* and *GWA2-50-13* are the two known anammox taxa within the analyzed datasets, where *Scalinduaceae* is globally present in deep-sea sediments (Figure 5) and both are associated with deep-sea environments (Sonthiphand et al., 2014; Zhao et al., 2022). *GWA2-50-13* is suggested to be part of *Bathyanammoxibiaceae*, which is widely distributed over sediments and soils (Zhao et al., 2022). Our results show that *Bathyanammoxibiaceae* is not as abundant as *Scalinduaceae* in the deep-sea sediments (Figure 5). Their dependence on ammonium and nitrite are consistent with our findings, as anammox appears in more reduced, oxygen-free environments, yet sufficiently oxidized to contain nitrite (Figure 3B). Anammox play a crucial role in the nitrogen cycle using their energy metabolism. Additionally, they might play a role in the cycling of the toxic nitric oxide intermediate (Johnston, 1971) through nitric oxide reduction (Kartal et al., 2007) to dinitrogen gas (N_2_) (Kartal et al., 2010). The nitrogen removal efficiency increased with an increased supply of nitric oxide by denitrifying microorganisms (Kartal et al., 2010; Hu et al., 2019; Zhang et al., 2024), bypassing the production of nitrous oxide from nitric oxide in the denitrification pathway and potentially suppressing the production of the greenhouse gas nitrous oxide.

### 3.6 Anaerobic heterotrophy

The widespread anaerobic chemoheterotrophs identified in this study include members of the phyla *Chloroflexota* (*Dehalococcoidia, GIF3*, and *Sh765B-AG-111*), *Planctomycetota* (*AKAU3564 sediment group* and *SG8-4*), *Desulfobacterota* (*Desulfosarcinaceae* and *Desulfatiglandaceae*), as well as the classes *Lokiarchaeia* and *JS1*, and order *Aerophobales*. These taxa were found to be abundant in anoxic sediments (Figure 3B, Supplementary Figures S5–S10). Below, we summarize the current knowledge regarding these widespread.

Members of the phylum *Chloroflexota* include many lineages that are abundant in anaerobic environments. Members of the class *Dehalococcoidia* are generally considered anaerobic (Hoshino et al., 2020), although this class also includes lineages that can grow aerobically (e.g., *SAR202* clade). Several isolated strains from *Dehalococcoidia* have been described as obligate organohalide-respiring bacteria (Löffler et al., 2013; Key et al., 2017). Notably, the strains exhibit a strictly hydrogenotrophic, organohalide-respiring metabolism, utilizing halogenated organic compounds as electron acceptors, and were shown to be non-motile (Löffler et al., 2013). Because the selected widespread taxon remains unclassified at lower taxonomic levels, its specific metabolic capacities cannot yet be resolved. Likewise for the orders *GIF3* (Begmatov et al., 2021; Wu et al., 2023) and *Sh765B-AG-111* (Vuillemin et al., 2020), that have been reported primarily from anoxic sediments (Löffler et al., 2013), but their metabolic potential remains poorly characterized.

The families *Desulfosarcinaceae* and *Desulfatiglandaceae* are known to reduce sulfate (Galushko and Kuever, 2021; Watanabe et al., 2017), where *Desulfatiglandaceae* can perform complete oxidation of recalcitrant and chlorinated hydrocarbons (Suzuki et al., 2014; Galushko and Kuever, 2021).

*Lokiarchaeia* is a widely observed archaeal lineage that has shown metabolic activity in Namibian shelf sediments, particularly under sulfur-reducing conditions. Gene expression patterns suggest anaerobic mixotrophic capabilities, allowing the utilization of sugars and amino acids, as well as carbon fixation via the Wood–Ljungdahl pathway (Orsi et al., 2020). To date, only one strain of *Lokiarchaeia* has been successfully cultered, namely *Promethearchaeum syntrophicum*. This strain was a non-motile chemoorganotroph that assimilates amino acids, peptides, and yeast extract. It depends on syntrophic interactions with hydrogen- or formate-utilizing microorganisms and does not use sulfate, thiosulfate, and nitrate as terminal electron acceptors (Imachi et al., 2024). The specific electron acceptor used by *P. syntrophicum* remains unidentified.

The *AKAU3564 sediment group* and *SG8-4* belong to the phylum *Planctomycetota*. Recently, the first cultured representative from the *SG8-4* lineage, *Anaerobaca lacustris* (family *Anaerobacaceae*), was isolated and characterized showing a strictly anaerobic heterotrophic metabolism with an unknown electron acceptor (Khomyakova et al., 2024). While the *AKAU3564 sediment group* is widely detected in deep-sea sediment sequence data, no further physiological or metabolic characterization is currently available.

The class *JS1* (renamed as *Candidatus Atribacteria*) and order *Aerophobales* have been repeatedly associated with gas hydrate-rich environments (Inagaki et al., 2006; Liu et al., 2019; Dong et al., 2019; Liu et al., 2022). Both groups have the genomic capacity to perform anaerobic degradation of hydrocarbons (Nobu et al., 2016; Liu et al., 2019; Dong et al., 2019) and often co-occur in anoxic marine sediments (Liu et al., 2022). High relative abundances of *JS1* and *Aerophobales* were detected at the anoxic WNAG site (Supplementary Figure S6), as well as in anoxic AMOR sediments at hydrothermal vent areas (GS21GC07 and GS21GC08) and nearshore sites with presumably elevated organic matter content (GS16GC05 and GS16GC06) (Supplementary Figure S5).

Despite advances in characterization of anaerobic hetetrotrophs in marine sediments, our understanding about their metabolic influence on the cycling of organic matter and their electron acceptors remains limited. For the class *Dehalococcoidia*, uncertainties remain regarding its role in degrading recalcitrant and chlorinated hydrocarbons and its use of electron acceptors. Similarly, the metabolic capacities and/or the potential electron acceptors of *Lokiarchaeia, GIF3, Sh765B-AG-111, AKAU3564 sediment group, SG8-4, JS1*, and *Aerophobales* are still poorly characterized. Expanding our knowledge in their metabolic capacities is essential for assessing their contributions to Earth's geochemical cycles.

### 3.7 Survival of strict anaerobes

Despite energy limitations (Hoehler and Jørgensen, 2013; LaRowe and Amend, 2015; Lever et al., 2015) and metabolic inhibition by oxygen (Lu and Imlay, 2021), the strict anaerobic widespread taxa showed a marked relative increase under favorable conditions when oxygen was depleted (Figure 4), implying that either (i) the anaerobes decay slower than the aerobic community or (ii) they grow faster. In core GS19GC10, the shift occurred roughly at 35,000 ± 900 years after seafloor colonization through burial, meaning that these anaerobic microorganisms may have endured oxic conditions for thousands of years. The assumption of these microorganisms having survived these conditions over a long time largely rests on the argument that deep-sea sedimentary microorganisms are considered immobile due to energy constraints, which prevents cell migration over depth (Hoehler and Jørgensen, 2013; Lever et al., 2015). This immobility is further supported by laboratory experiments showing no indications of motility capabilities on strains commonly found in sediments, such as in the *Desulfatiglandaceae* (Galushko and Kuever, 2021) and *Desulfatiglans* families (Suzuki et al., 2014).

The viability of anaerobes after long-term dormancy is remarkable when considering 35,000 years of exposure to reactive oxygen species (ROS). ROS, such as superoxide and hydrogen peroxide, are highly reactive with DNA, RNA, proteins, and lipids and can cause vital damage without a defense mechanism. Aerobic metabolisms fuel the production of ROS by respiration, where both aerobes and anaerobes are equipped with protective mechanisms against ROS (reviewed in Johnson and Hug, 2019). For example, the non-spore forming *Dehalococcoides* spp. (Löffler et al., 2013) can withstand oxygen exposure (Amos et al., 2008; Yoshikawa et al., 2017). However, during an oxygen exposure experiment on *Dehalococcoides* from anaerobic aquifer sediments, prolonged exposure of oxygen led to loss of viability after 30 days. Moreover, subsequent change of the experiment to anaerobic conditions for up to 262 days did not reveal any signs of activity (Amos et al., 2008). Therefore, the survival of anaerobes without the ability to produce energy for thousands of years, while maintaining ROS defenses and damage repair, seems unrealistic in this regard. This leads to a series of important questions on whether other mechanisms are present in deep-sea sediments that lead to the viability of anaerobes after 35,000 years. Do anaerobes in the deep-sea have more energy efficient ROS defenses? Have deep-sea anaerobes evolved to be less susceptible to oxidative stress through spore formation (Setlow, 1995)? Do ROS defense mechanisms of aerobes contribute to the survival of anaerobes? Or does the low activity of aerobes result in significantly less production of ROS, and therefore, less exposure of anaerobes to ROS? As alternatives to a biological factor, the (i) ROS diffusing out of oxygen respiring cells can be chemically scavenged by abiotic reactions of reduced substances, such as ferrous iron (Adjimani and Asare, 2015; Neubeck and Freund, 2020). Or (ii) the surviving anaerobes may have resided in anoxic microniches as shown in coastal marine sediments (Jørgensen, 1977; Lehto et al., 2014; Jalaluddin et al., 2025). The scavenging, lower production of ROS, and anoxic microniches might promote the survival of anaerobes. The viability of these microorganisms remains to be further investigated. Transcriptomics, proteomics, or stable isotope probing experiments are required to gain further insights into the activities of anaerobes in the oxic zone after thousands of years of survival.

### 3.8 Relevance to global carbon cycling

Widespread microorganisms influence the marine carbon cycle through their autotrophic and heterotrophic metabolisms. In this section, we discuss the relevance of these metabolic pathways to the carbon cycle according to literature.

Deep-sea sediments serve as a long-term sink for carbon through the burial of calcium carbonate and organic matter, with an estimated accumulation rate of 0.15–0.2 Pg C yr^−1^ (Hedges and Oades, 1997; Sarmiento and Gruber, 2002; Hayes et al., 2021). Within the seafloor, the carbon cycling continues through both biotic and abiotic processes. The biotic impact on carbon burial through sequesteration and degradation is less well understood. Our findings highlight the importance of both autotrophs and heterotrophs in shaping geochemical gradients and therefore carbon turnover (Figure 3B; Sections 3.3–3.6). Autotrophs sequester carbon through inorganic carbon fixation to form organic carbon, whereas heterotrophs remineralize organic carbon. Therefore, inorganic carbon fixation is suggested to sustain the food web in the deep-sea surface sediments (Molari et al., 2013). Additionally, heterotrophs are suggested to incorporate dissolved inorganic carbon via anaplerotic reactions that replenishes intermediates for the tricarboxylic acid (TCA) cycle (Krebs, 1941; Kornberg, 1965; Braun et al., 2021). Yet, the extent of their ability to fixate inorganic carbon is not well understood.

Microorganisms involved in aerobic processes, particularly facultative anaerobic heterotrophs and nitrifiers (Sections 3.3–3.4), are thought to contribute the most to carbon cycling in deep-sea sediments (Middelburg, 2011). Heterotrophs belonging to sediments from seafloor where the water depth is 2,000 m or more are estimated to degrade 0.66-0.85 Pg C yr^−1^ through respiration (55–71 Tmol C yr^−1^; Jørgensen et al., 2022). Nitrifiers, specifically ammonium oxidizers, such as the widespread *Nitrosopumiales* (Section 3.3), sequester 0.004 Pg C yr^−1^ through inorganic carbon fixation (Middelburg, 2011). Ammonium oxidizers are assumed to be the main contributors to inorganic carbon fixation among chemoautotrophs in deep-sea sediments (Middelburg, 2011). This assumption is supported by the high absolute abundance of crenarchaeotal 16S rRNA and Amoa genes estimated through quantitative PCR (Wuchter et al., 2006) and ^13^C labeling experiment, showing nitrifiers as the main contributors to inorganic carbon fixation in the upper 5 cm of sediments (~1280 m water depth) in the Fram Strait (Guilini et al., 2010). Consistent with these findings, our results show that nitrifiers (*Nitrosopumilaceae* and *Nitrospinaceae*) and putative denitrifiers (*Kiloniellaceae, Woeseiaceae, SAR202* clade, *JTB23, Defluviicoccales*, and *Magnetospiraceae*) predominate in the oxic sediments (Figure 4, Supplementary Figures S5–S10; Section 3.3, 3.4). Nonetheless, high microbial abundance based on DNA does not directly imply high metabolic activity (Zakem et al., 2022), marking the need for activity-based approaches, such as transcriptomics or isotopic labeling.

The contribution of anaerobic processes to carbon cycling in deep-sea sediments remains underrepresented in global assessments. Although, some global carbon rates are reported for anaerobic processes, these estimates do not include all biogeochemical processes microorganisms influence. Our results suggest that anammox bacteria are key players in the cycling of carbon and nitrogen at the oxic-anoxic transition zone (Figure 3B). Globally, anammox have been estimated to fix 0.00017–0.0035 Pg C yr^−1^ (Raven, 2009), which is two to three orders of magnitude lower than the oceanic carbon fixation by aerobic ammonium oxidizers (0.77 Pg C yr^−1^; Middelburg, 2011). However, the direct comparison of ammonium oxidizers and anammox on a global scale does not translate to local ecological importance. Anammox activity is limited to niches where both nitrite and ammonium co-occur, creating smaller areas of activity than aerobic ammonium oxidizers. Aerobic ammonium oxidizers dominate more area due to the more widespread co-occurence of ammonium and oxygen. Anammox plays a crucial role in oxygen minimum zones by increasing nitrogen loss efficiency and reducing greenhouse gas emissions of nitrous oxide (Kartal et al., 2010), which will be further discussed in Section 3.9.

The results show that anaerobic heterotrophs also appear to play an important role in deep-sea carbon cycling, but are largely undercharacterized. The electron acceptors used by the widespread microorganisms remain frequently unidentified (Section 3.6), making it difficult to establish their metabolic contributions to the carbon cycle. The contribution to the carbon cycle is often assessed using mineralization ratio based on the electron acceptor, requiring balanced chemical equations (Jørgensen, 2021; Jørgensen et al., 2022). Consequently, their quantitative contribution to carbon degradation remains largely unknown. Among anaerobic heterotrophs, only sulfate reduction has published estimates of carbon turnover. Widespread taxa, such as *Desulfosarcinaceae* and *Desulfatiglandaceae*, contribute to sulfate reduction in deep-sea sediments (Section 3.6). Based on sulfate reduction estimates in the continental rise and abyss (>2000 m depth, total of 1.44 Tmol sulfate (${\text{SO}}_{4}^{2-}$) yr^−1^; Canfield et al., 2005 reviewed in Jørgensen, 2021) the carbon degradation rate by sulfate reducers is 0.029 Pg C yr^−1^ when considering a CHO_2_:${\text{SO}}_{4}^{2-}$ mineralization ratio of 1.7:1 (Jørgensen, 2021). In contrast, other anaerobic metabolisms, such as iron oxide and manganese oxide reduction coupled to mineralization of organic matter, lack published turnover estimates. These may represent potential electron acceptors for the uncharacterized heterotrophic anaerobic widespread taxa. The identification of genes related to iron cycling has been recently more established (Garber et al., 2020), whereas manganese oxide reduction has until now only been empirically defined, as for example by (Leu et al. 2020).

Further research is needed to quantify inorganic carbon fixation via additional pathways, including the oxidation of sulfur compounds, carbon monoxide (CO), methane (CH_4_), as well as determine the potential contribution of heterotophs through anaplerotic reactions (reviewed in Braun et al., 2021). Likewise, the quantification of the heterotrophic organic matter degradation using alternative electron acceptors, such as iron oxide, manganese oxide, and reduction of intermediates such as thiosulfate remains to be explored. More knowledge into these metabolic pathways is required to obtain carbon degradation rate estimates using reactive transport modeling or isotopic tracing experiments. Moreover, there is limited knowledge on spatial variability of carbon cycling rates across deep-sea environments. For example, inorganic carbon fixation by sulfide oxidizing bacteria within hydrothermal vents systems (0.002 Pg C yr^−1^; Raven, 2009) and basaltic ocean crust (0.001 Pg C yr^−1^; Bach and Edwards, 2003) were found to contribute 0.003 Pg C yr^−1^. These sulfide oxidizers in this area sequesters an equivelent of 75% of the estimated carbon sequestered by aerobic ammonium oxidizers in the deep-sea sediments. This emphasizes the need to characterize the spatial variability of carbon cycling rates across deep-sea environments to refine global deep-sea carbon budgets.

While 16S rRNA gene data and pore water geochemistry (i.e., oxygen, nitrate, ammonium, and manganese) can reveal taxa correlated with redox changes, they do not provide enough evidence to infer organism-specific chemical transformations. 16S rRNA gene-based classification often lacks the taxonomic resolution needed to distinguish closely related lineages and cannot directly determine metabolic function. Robust functional inference therefore requires metagenomic and metatranscriptomic approaches, which can link taxa to metabolic potential and whether the genes are expressed *in situ*. Furthermore, omitting other key redox-sensitive constituents, such as sulfate, sulfite, and iron- and manganese oxides, etc., can underestimate the importance of microorganisms that use these electron acceptors during organic matter degradation. Therefore, more research is required toward other important redox-sensitive constituents and the characterization of the metabolic potential of uncharacterized lineages.

### 3.9 Environmental perturbations

Widespread microorganisms may serve as important indicators of climate change and provide early evidence of shifts in ecosystem functioning under environmental perturbations. Changes in ocean's physical and chemical properties are known as important drivers of microbial activity (Danovaro et al., 2001; Yasuhara and Danovaro, 2016; Sweetman et al., 2017). Alterations in temperature, dissolved oxygen, and chemical composition (Breitburg et al., 2018), as well as anthropogenic disturbances such as deep-sea mining of Mn-nodules (Vonnahme et al., 2020), can disrupt the balance of microbial processes and, consequently, the climate feedback system. Yet, the response of deep-sea sediment microbial communities to such perturbation remains poorly understood, including changes in microbial community structure, metabolic functioning, and the recovery rates. For instance, a study on the effect of nodule mining on the microbial community revealed that 26 years was insufficient to restore the initial functioning of the community once disturbed, due to low current speeds, low sedimentation rates, and low bioturbation (Vonnahme et al., 2020). Microbial and bioturbation activities were substantially reduced at highly disturbed sites leading to significantly lower rates of carbon dioxide (CO_2_) fixation and dissolved oxygen uptake (Vonnahme et al., 2020). Forty-four years after small-scale nodule mining activities, the micro and macrofauna started to reestablish in the area, except for directly disturbed areas (Jones et al., 2025). This demonstrates the impact disruption can have on the ecosystem and geochemical cycles.

Understanding the change in biogeochemical cycles due to climate change is important to understand the future Earth's climate conditions and habitability. The primary biotic processes in the sediments influencing the carbon cycles are carbon dioxide fixation, organic matter degradation, and methane formation. However, the formation of methane through methanogenesis typically represents a minor carbon-cycling pathway in deep-sea sediments (Egger et al., 2018), since methanogenesis is primarily driven by labile organic matter load (Naqvi et al., 2010). Overall, organic matter load is predicted to decrease in the year 2100, except for (i) the polar ocean due to the retreat of sea ice and (ii) deep seafloor regions near upwelling zones with increased nutrient upwelling caused by increased wind stress (Sweetman et al., 2017). Reduced primary productivity may result in less carbon burial and life in deep-sea sediments, which could be observed as a decrease in the widespread heterotrophic microorganisms.

Projected changes in ocean physico–chemical conditions might change the balances in Earth's nitrogen cycle. Currently, oceans are experiencing a decline in oxygen concentrations due to rising temperatures, which reduces the solubility of oxygen (Oschlies et al., 2018; Breitburg et al., 2018). This event can lead to the expansion of oxygen minimum zones that enhance nitrous oxide production, a known greenhouse gas (Naqvi et al., 2010). Nitrous oxide is produced as an intermediate by nitrifiers (Voland et al., 2025) and denitrifiers (Ji et al., 2018), such as the putative contributors found in this study (Sections 3.3, 3.4, respectively). The consequences can potentially be mitigated by the presence of anammox (Hu et al., 2019), another functional group found in our study (Section 3.5). Anammox has been shown to detoxify the nitric oxide pollutant (Johnston, 1971) through reduction to dinitrogen gas, deminishing the conversion of nitric oxide to nitrous oxide by other denitrifying bacteria (Kartal et al., 2010; Hu et al., 2019). This nitric oxide scavenging highlights the importance of the entire community, from heterotrophic denitrifiers to autotrophic nitric oxide reduction in contributing to nitrogen loss with minimal greenhouse gas emissions. An imbalance in anammox versus denitrifing bacteria ratio can promote nitrous oxide accumulation (Kartal et al., 2010), which might become problematic in expanding oxygen minimum zones in both the sediments and the water column.

More research is needed to resolve microbial community's response to various climate scenarios and how shifts in microbial communities impact global biogeochemistry, for example through greenhouse gas production and consumption and the burial of carbon (Zakem et al., 2020). Improved understanding of these processes is essential for predicting the future stability and functioning of deep-sea ecosystems with increasing environmental perturbations. The widespread microorganisms identified in this study provide a useful starting point for prioritizing taxa that are most likely (i) to advance our understanding of deep-sea sediment contributions to carbon and nitrogen cycle fluxes, (ii) serve as indicators of environmental change, and (iii) help evaluate how shifts in microbial community structure alter the balance among biogeochemical pathways that regulate net greenhouse gas uptake or emission. However, we note that these taxa are not the only ones that should be considered. The set of widespread microorganisms identified here is constrained by the reliance on 16S rRNA gene data and by the limited number of redox-sensitive constituents used to characterize the environment. More widespread taxa will likely be revealed by metagenomic approaches, which can mitigate primer bias, improve taxonomic resolution, and enable more direct inference of metabolic potential. In addition, more redox-sensitive constituents (e.g., iron oxide, manganese oxide, sulfate, sulfite, etc.) play and important role in deep-sea sediments carbon and nitrogen cycles. Therefore, more redox-sensitive constituents should be considered to obtain a complete picture of how deep-sea sedimentary microorganisms influence biogeochemical cycles.

## 4 Conclusions

We identified known and unknown widespread taxa in deep-sea sediments that can have a pronounced influence of global geochemical cycles. The taxa related to four main types of metabolisms: (1) aerobic nitrification, (2) facultative anaerobic heterotrophy, (3) anaerobic ammonium oxidation, and (4) anaerobic heterotrophy. Their distribution appears to be strongly influenced by redox potential that might be affected by future climate changes with reduced dissolved oxygen in the ocean and direct ecosystem perturbation, such as deep-sea mining, which will change ecosystem functioning. These microorganisms might serve as indicators for environmental change and enhance our knowledge of important processes to global climate cycles, which improves the current oversimplified presentation of microbial activity in Earth system models. However, more research is needed to identify other widespread deep-sea sedimentary microorganisms to have a complete overview of the important taxa influencing global biogeochemical cycles. Integrating metagenomics, metatranscriptomics, and isotopic labeling experiments would help identify additional taxa, link them to metabolic potential, and quantify their activity under *in situ* conditions.

## Data Availability

The data presented in this study are publicly available. The raw sequencing data can be found in the NCBI repository (https://www.ncbi.nlm.nih.gov/) under the accession numbers listed in Table 1. The chemical datasets are available in the PANGAEA database (Hageman et al., 2026; Brendryen et al., 2026). The code is available in the github repository via the following link: https://github.com/The-deep-biosphere/2026-Widespread_deep-sea_sedimentary_microorganisms.
