# Supplementary material for: Widespread deep-sea microorganisms in subseafloor geochemical cycling
Source: Front Microbiol. 2026 Apr 15;17:1790163. doi: 10.3389/fmicb.2026.1790163 (PMC13127444; doi:10.3389/fmicb.2026.1790163)
Supplement: Supplementary file 1 [file Data_Sheet_1.pdf]

Supplementary Material

1 SUPPLEMENTARY TABLES AND FIGURES

1.1 Figures

1.1.1 Model Performance

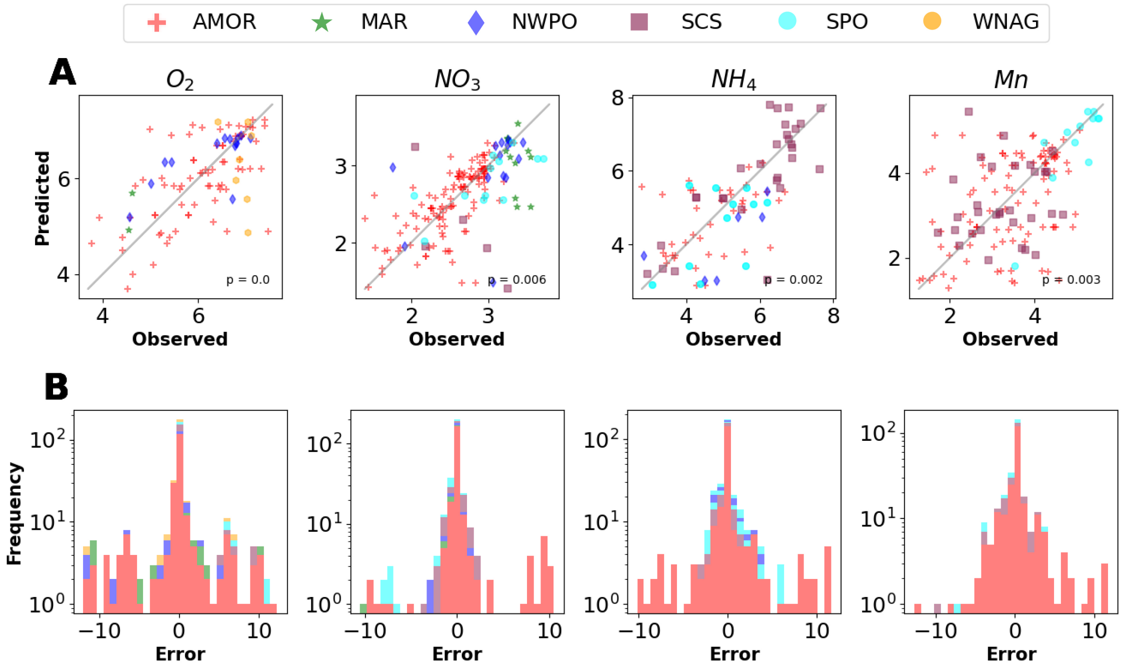

Figure S1. Decision tree regressor prediction of oxygen, nitrate, ammonium, manganese trained on all locations. (A) Prediction of clr transformed chemical concentrations based on microbial community data. The gray line is the 1:1 line that would be the perfect prediction. Only concentrations higher than zero are shown. p indicates the p-value of the datapoints depicted calculated using scipy.stats.pearsonr in $Python$. (B) Histrogram with log transformed frequency and clr transformed error distribution including all predictions.

1.1.2 Selected taxa overview

Decision Tree Regressor

Presence

	O_2	NO_3	NH_4	Mn	Number of Samples
AMOR	88.2	91.2	60.0	96.1	93/148/60/152
WNAG	80.0	nan	nan	nan	10/0/0/0
MAR	50.0	100.0	nan	nan	6/8/0/0
NWPO	90.0	85.7	66.7	nan	20/21/6/0
SPO	nan	27.3	100.0	94.4	0/11/15/18
SCS	nan	50.0	94.9	100.0	0/8/39/32

Absence

	O_2	NO_3	NH_4	Mn	Number of Samples
AMOR	88.3	86.5	93.9	77.5	137/74/165/80
WNAG	100.0	nan	nan	nan	14/0/0/0
MAR	33.3	nan	nan	nan	3/0/0/0
NWPO	66.7	87.5	84.6	nan	3/8/13/0
SPO	90.9	94.1	42.9	75.0	11/17/7/8
SCS	93.3	86.7	0.0	nan	30/30/5/0

Decision Tree Classifier

Presence

	O_2	NO_3	NH_4	Mn	Number of Samples
AMOR	85.2	90.7	62.9	97.0	93/148/60/152
WNAG	87.4	nan	nan	nan	10/0/0/0
MAR	83.3	75.7	nan	nan	6/8/0/0
NWPO	91.3	85.7	78.0	nan	20/21/6/0
SPO	nan	94.8	91.4	98.7	0/11/15/18
SCS	nan	38.2	95.7	96.9	0/8/39/32

Absence

	O_2	NO_3	NH_4	Mn	Number of Samples
AMOR	90.8	82.3	93.1	94.8	137/74/165/80
WNAG	100.0	nan	nan	nan	14/0/0/0
MAR	29.2	nan	nan	nan	3/0/0/0
NWPO	51.4	86.7	87.0	nan	3/8/13/0
SPO	84.1	94.0	60.5	78.0	11/17/7/8
SCS	94.2	88.2	7.7	nan	30/30/5/0

Figure S2. DT regressor and classifier accuracy comparison for the presence and absence of each constituent for every location. The number of samples shows how many samples are used on average for the consituent $O_2/NO_3^-/NH_4^+/Mn^{2+}$. The accuracy is the mean over 1000 independent iterations of DT training.

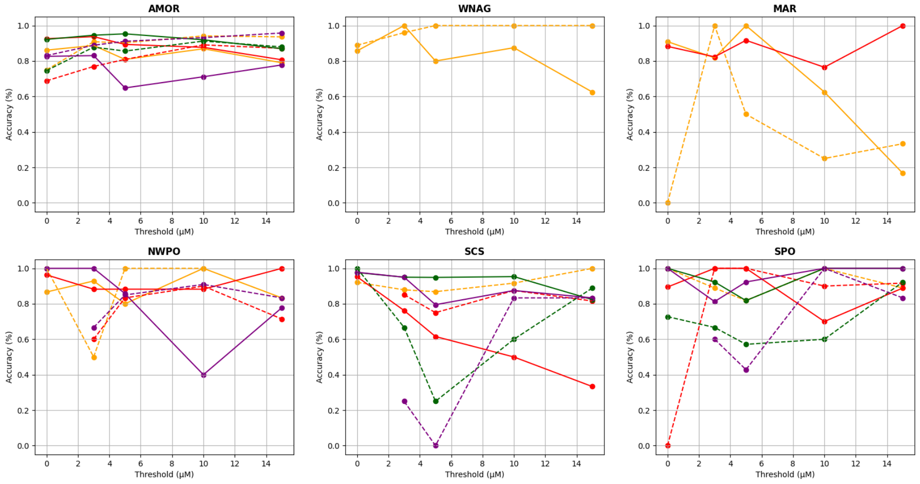

Figure S3. Sensitivity analysis on changing threshold of absence (dashed) or presence (line) of the chemical consituents (x-axis) plotted against the resulting prediction accuracy (y-axis). Orange is O_2, red is NO_3^-, dark green is Mn^{2+}, and purple is NH_4^+.

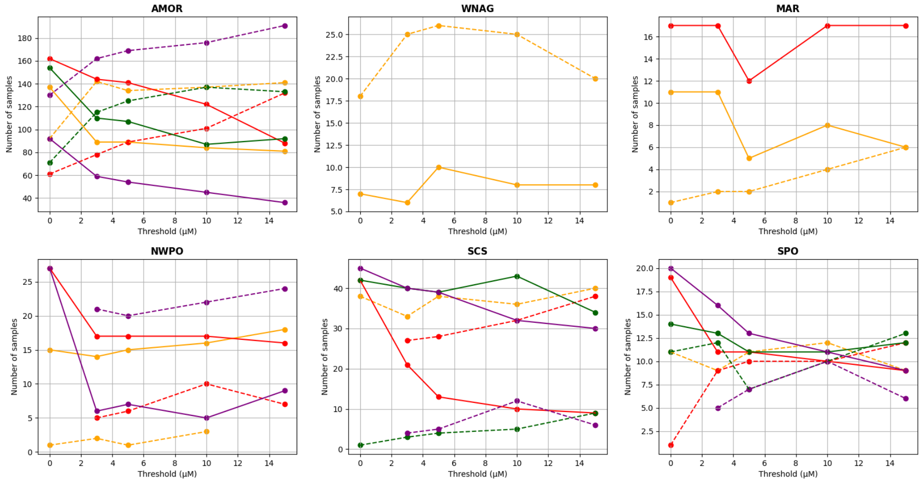

Figure S4. Sensitivity analysis on changing threshold of absence (dashed) or presence (line) of the chemical consituents (x-axis) plotted against the number of samples used to predict the accuracy in Figure S3 (y-axis). Orange is O_2, red is NO_3^-, dark green is Mn^{2+}, and purple is NH_4^+.

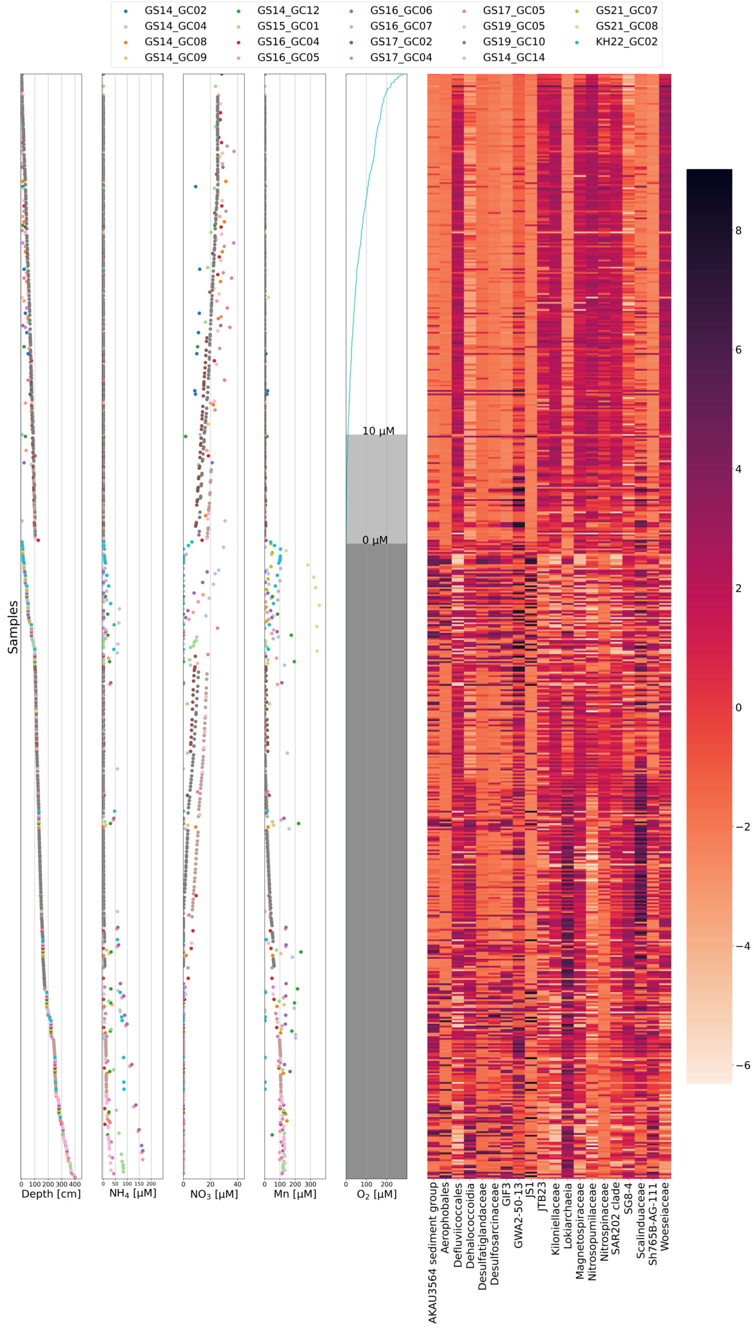

Figure S5. Overview of AMOR with oxygen and available nitrate, ammonium and manganese measurements. The relative abundances are clr-transformed. The data are ordered by descending oxygen concentration and therefore oxygen is displayed as a line. On the left side of the heatmap environmental variables (depth, ammonia, nitrate, manganese, and oxygen) are displayed.

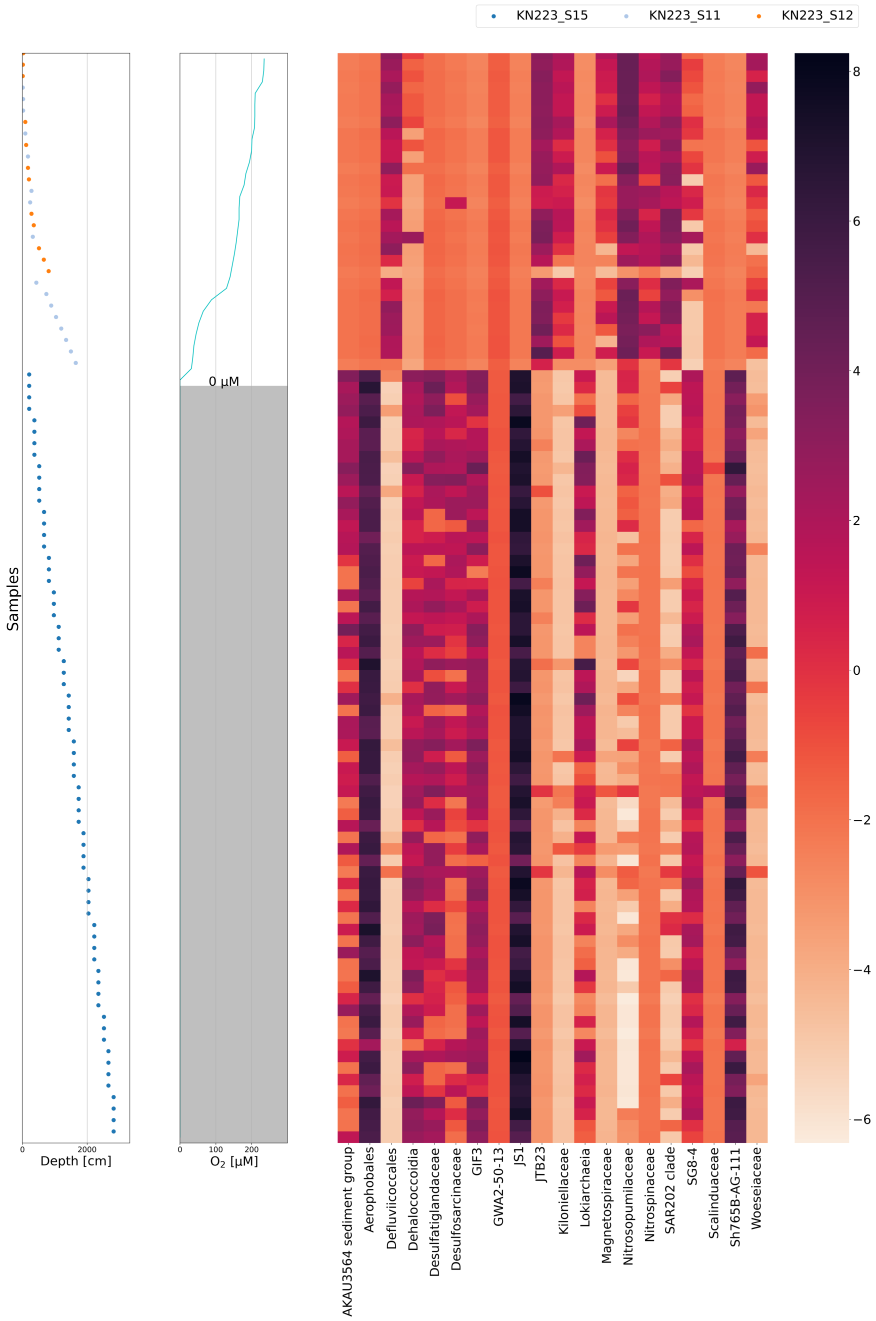

Figure S6. Overview of WNAG with oxygen measurements. The relative abundances are clr-transformed. The data are ordered by descending oxygen concentration and therefore oxygen is displayed as a line. On the left side of the heatmap environmental variables (depth and oxygen) are displayed.

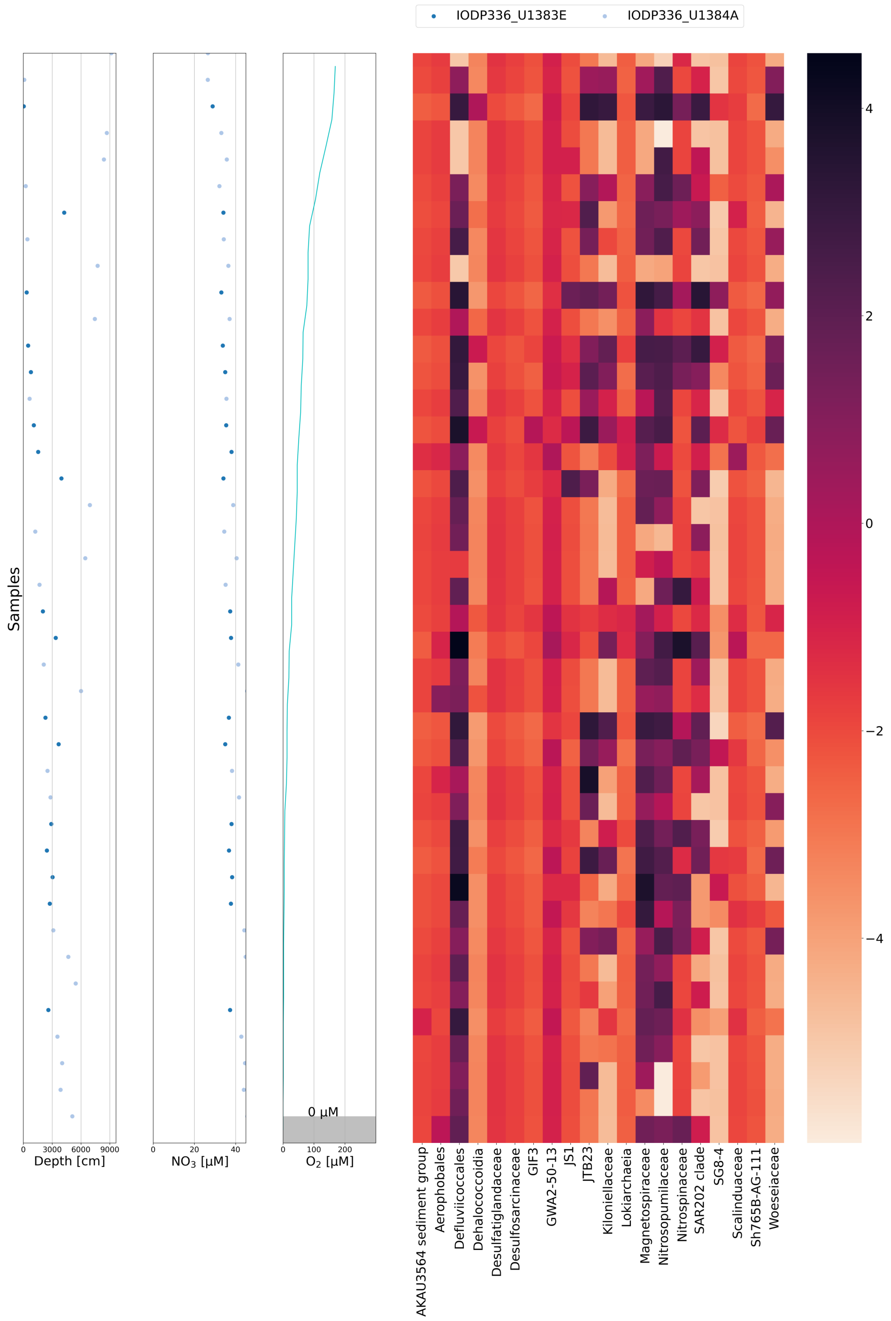

Figure S7. Overview of MAR with oxygen and nitrate measurements. The relative abundances are clr-transformed. The data are ordered by descending oxygen concentration and therefore oxygen is displayed as a line. On the left side of the heatmap environmental variables (depth, nitrate, and oxygen) are displayed.

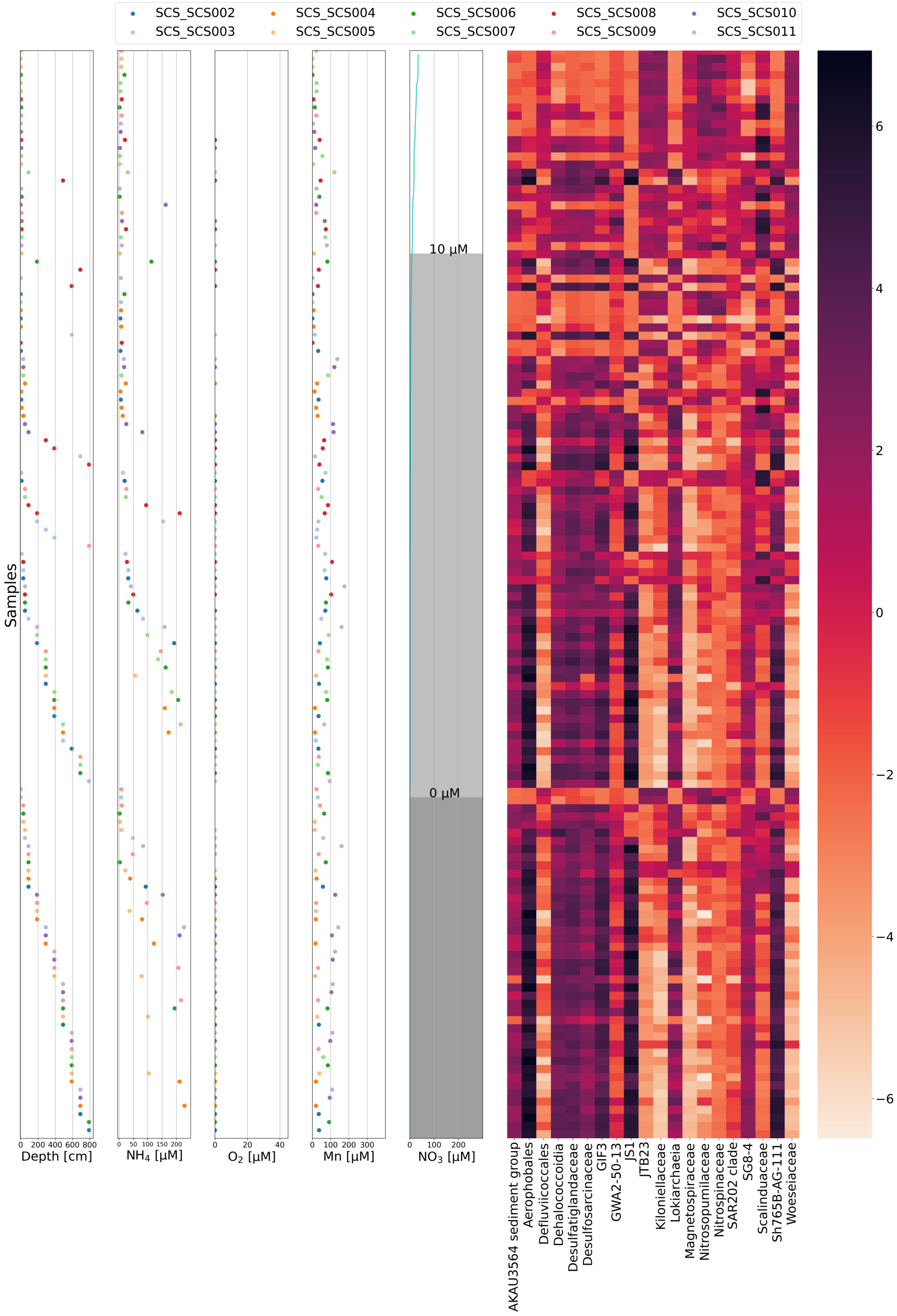

Figure S8. Overview of anoxic samples of SCS with available nitrate, ammonium and manganese measurements. The relative abundances are clr-transformed. The data are ordered by descending nitrate concentration and therefore nitrate is displayed as a line. On the left side of the heatmap environmental variables (depth, ammonia, oxygen, manganese, and nitrate) are displayed.

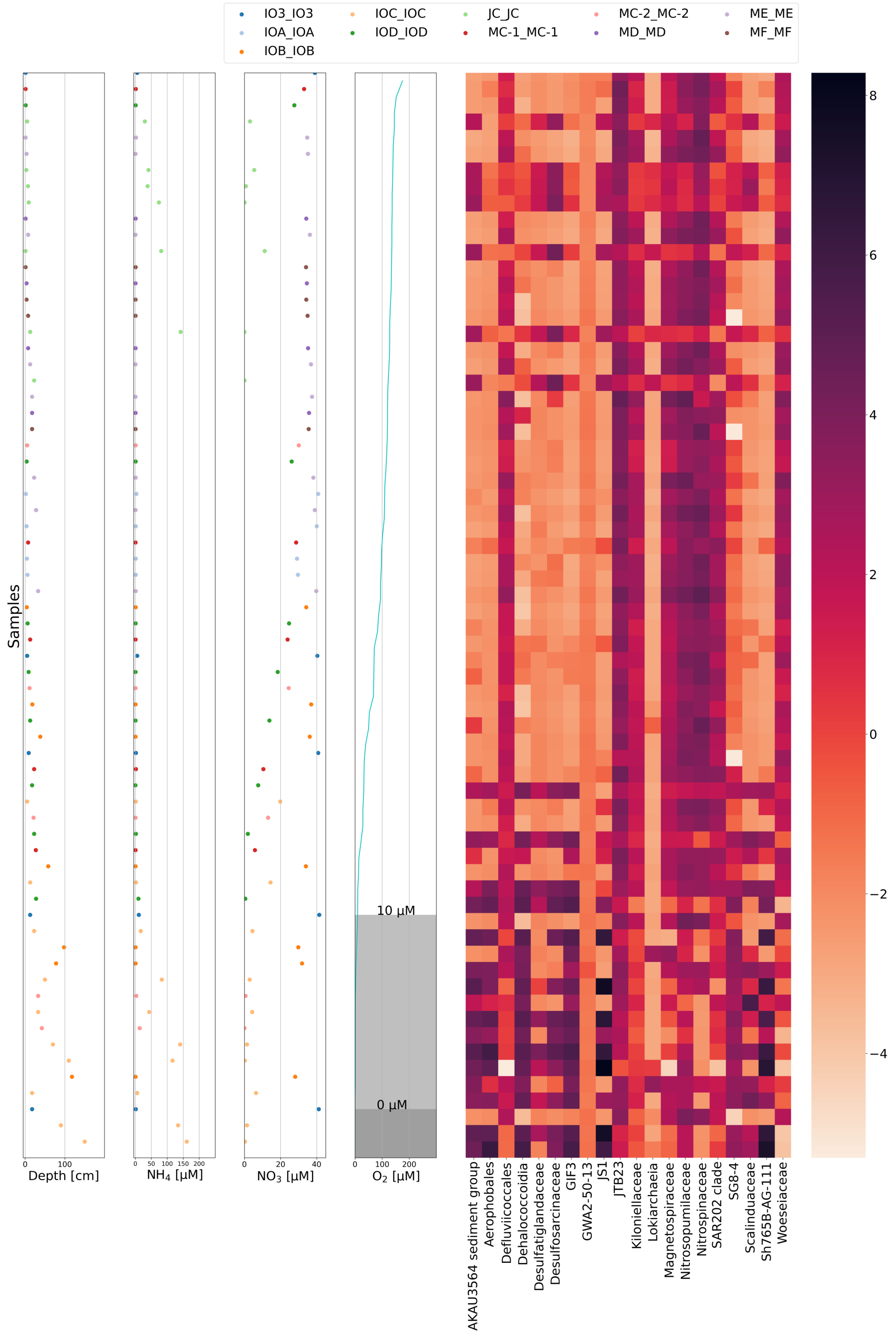

Figure S9. Overview of NWPO of oxygen with available nitrate and ammonium measurements. The relative abundances are clr-transformed. The data are ordered by descending oxygen concentration and therefore oxygen is displayed as a line. On the left side of the heatmap environmental variables (depth, ammonia, nitrate, and oxygen) are displayed.

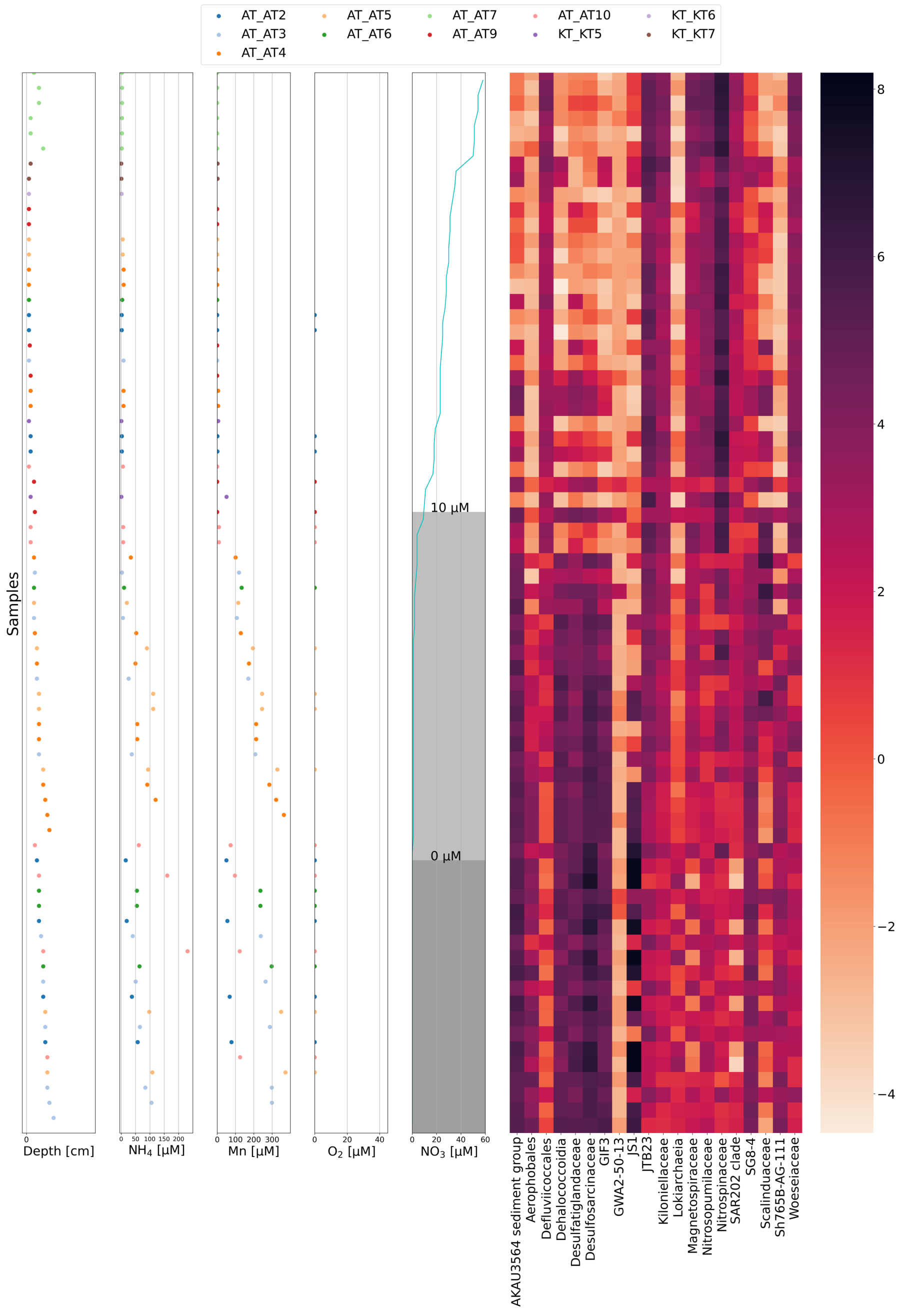

Figure S10. Overview of SPO of oxygen with available nitrate, ammonium and manganese measurements. The relative abundances are clr-transformed. The data are ordered by descending nitrate concentration and therefore nitrate is displayed as a line. On the left side of the heatmap environmental variables (depth, ammonia, oxygen, manganese, and nitrate) are displayed.
